# Late lower extremity free flap vascular compromise and salvage in a Pediatric patient diagnosed with monophasic synovial sarcoma

**DOI:** 10.1080/23320885.2023.2249092

**Published:** 2023-08-22

**Authors:** Shervin Zoghi, Kelsey Millar, Steven Thorpe, Christopher O. Bayne

**Affiliations:** Department of Orthopedic Surgery, UC Davis Health, Sacramento, CA, United States

**Keywords:** Flap failure, sarcoma, vascular compromise, flap salvage, pediatric orthopedic surgery

## Abstract

Free tissue flap transfer can be utilized for reconstruction following tumor resection. While flap failure occurs primarily within 72 h post-operation, late failure after day 7 is rare. We present the case of a 14-year-old with a late lower extremity free flap vascular compromise, along with the successful flap salvage.

## Introduction

Free tissue transfer is commonly utilized for reconstruction following lower extremity tumor resection. Approximately five percent of lower extremity flaps fail, primarily within 72 h post-operation due to thrombosis of the vascular pedicle [1]. However, failure of a flap after postoperative day 7 is rare because of the decreased reliance of the flap on the pedicle vessels [1]. While some researchers believe the cause to be excess pressure on the pedicle or residual tumor growth [2], the etiology and mechanism of late flap failure are still not well understood.

## Case presentation

A 14-year-old female patient presented to the orthopedic oncology clinic with a history of an unplanned excision of a monophasic synovial sarcoma located in the lateral ankle with positive margins. After arrival at our institution and review of the case with our multidisciplinary sarcoma board, the patient elected to proceed with limb salvage treatment with preoperative radiation therapy of 50 Gy in 25 fractions, followed by tumor bed excision and staged soft tissue reconstruction of the ankle.

**Figure 1.**
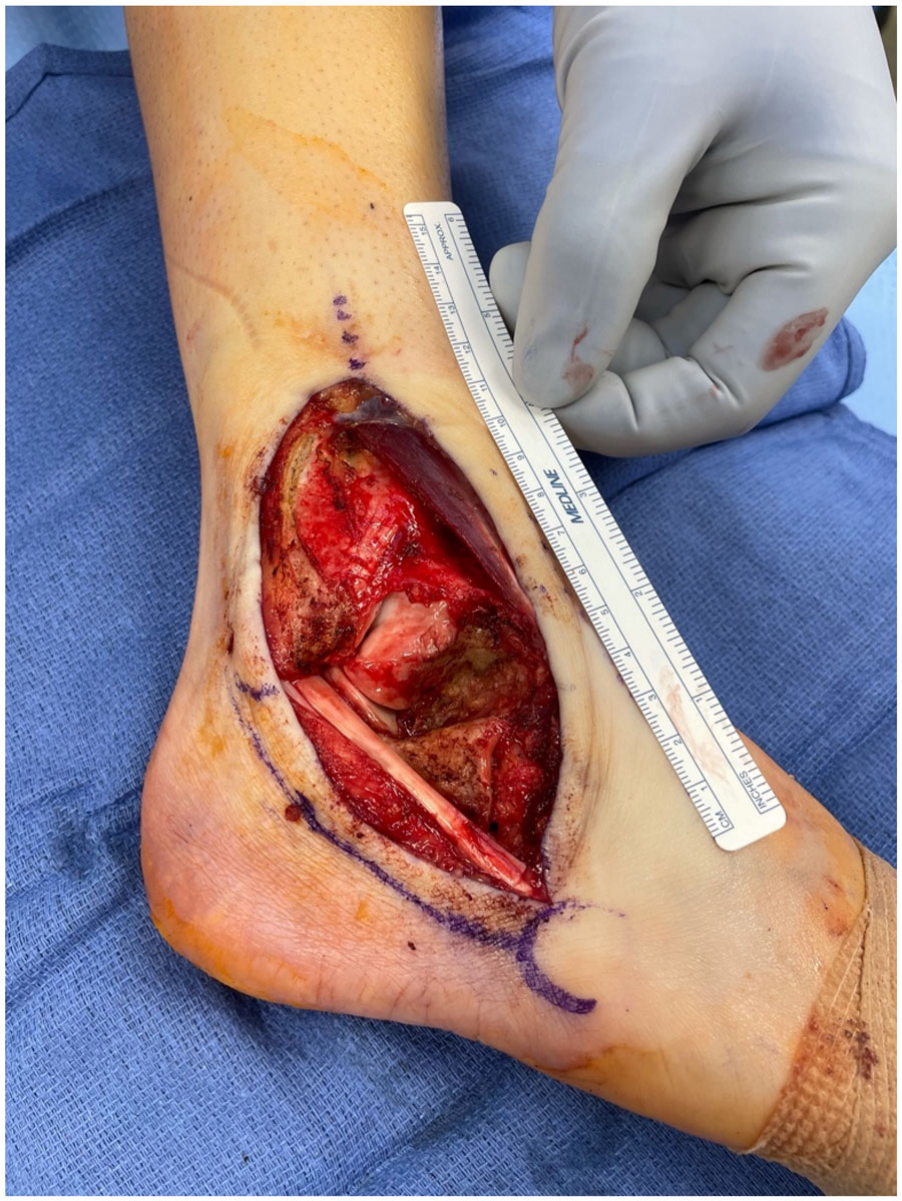
Pre-radial forearm flap soft tissue defect.

**Figure 2.**
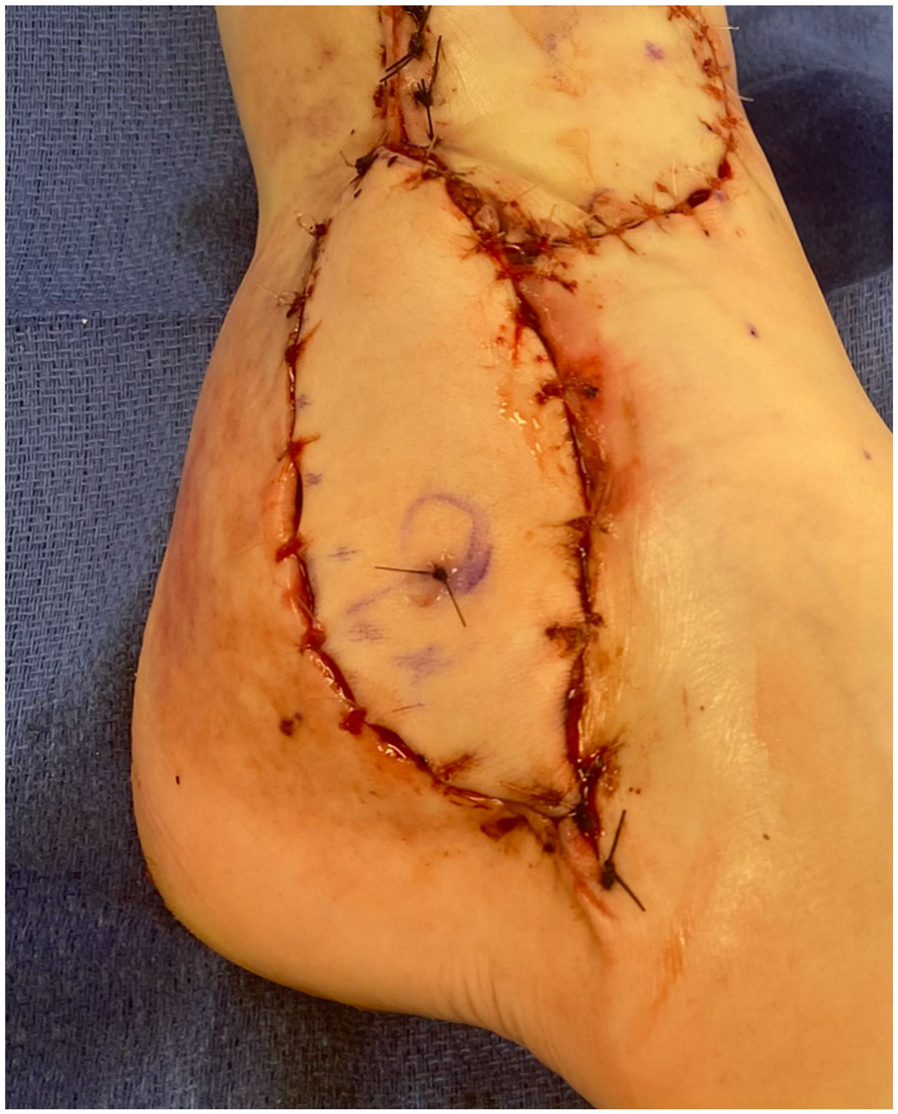
Right lower extremity wound appearance after radial forearm free flap. The proximal and distal portions of the defect were approximated with 4-0 Nylon sutures in a horizontal mattress fashion. The flap was inset with interrupted, simple 3-0 Monocryl sutures. The incision over the recipient anterior tibial artery was closed with interrupted, simple 3-0 Monocryl sutures. Nylon suture in the free flap marks the site at which doppler monitoring was performed over the flap postoperatively.EI

The patient had a defect in her lower lateral leg and ankle measuring 12 cm by 5 cm with exposed peroneal tendons and anterior tibia and talus with minimal overlying periosteum and joint capsule (Figure 1). Even following soft tissue mobilization, given the size of the defect and the associated adiposity of other potential donor sites, the decision was made to perform a radial forearm fasciocutaneous free flap reconstruction. The radial forearm free flap measured 9 cm x 4 cm. An incision was made over the distal aspect of the anterior leg to dissect the recipient vessel (the anterior tibial artery). This incision was connected to the defect to allow for anastomosis without subcutaneous tunneling. The radial artery pedicle was anastomosed to the anterior tibial artery. The largest radial artery venae commitment was anastomosed to the largest venae comitant of the anterior tibial artery, and the cephalic vein was anastomosed to a large superficial vein of the anterior leg. A Doppler flow monitor probe was placed at the anastomoses site. The proximal and distal portions of the defect were approximated with 4-0 Nylon sutures in a horizontal mattress fashion and the flap was inset with interrupted simple 3-0 Monocryl sutures. The incision over the recipient's anterior tibial artery was closed with interrupted, simple 3-0 Monocryl sutures (Figure 2). A windowed short-leg splint was placed to allow for visual monitoring.

Postoperatively, the patient was admitted to the Pediatric Intensive Care Unit (PICU) for frequent neurovascular flap checks and placed on a post-operative bed rest protocol. There were no acute postoperative complications and on postoperative day 6, the patient was transferred to floor-level of care. A progressive lower extremity dangle protocol was started. On postoperative day 9, the patient was noted to have worsening flap congestion, no capillary refill, acute loss of Doppler signal, and no bleeding following the puncture of the skin. Given the concern for flap failure, the patient was emergently taken to the operating room for flap exploration.

While there was no visible or doppler able flow across the arterial or venous anastomoses, there was no visible thrombosis. The anastomoses were resected, and all flap and recipient vessels were irrigated with heparinized saline. Balloon embolectomy catheters were placed across all vessel stumps without significant thrombosis removed. Alteplase was irrigated through the flap arterial pedicle.

**Figure 3.**
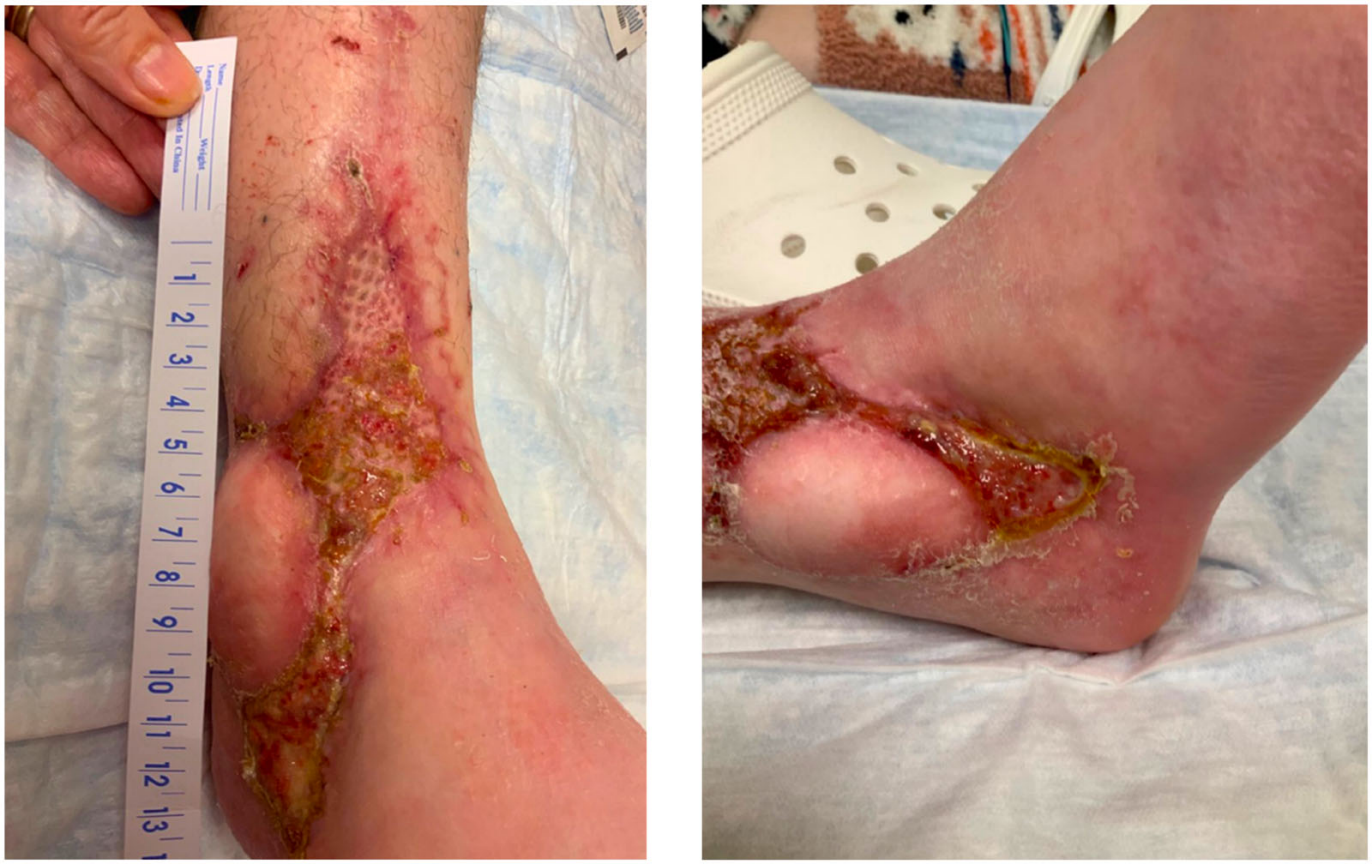
Right lower extremity 4-months postoperatively after flap salvage procedure.

**Figure 4.**
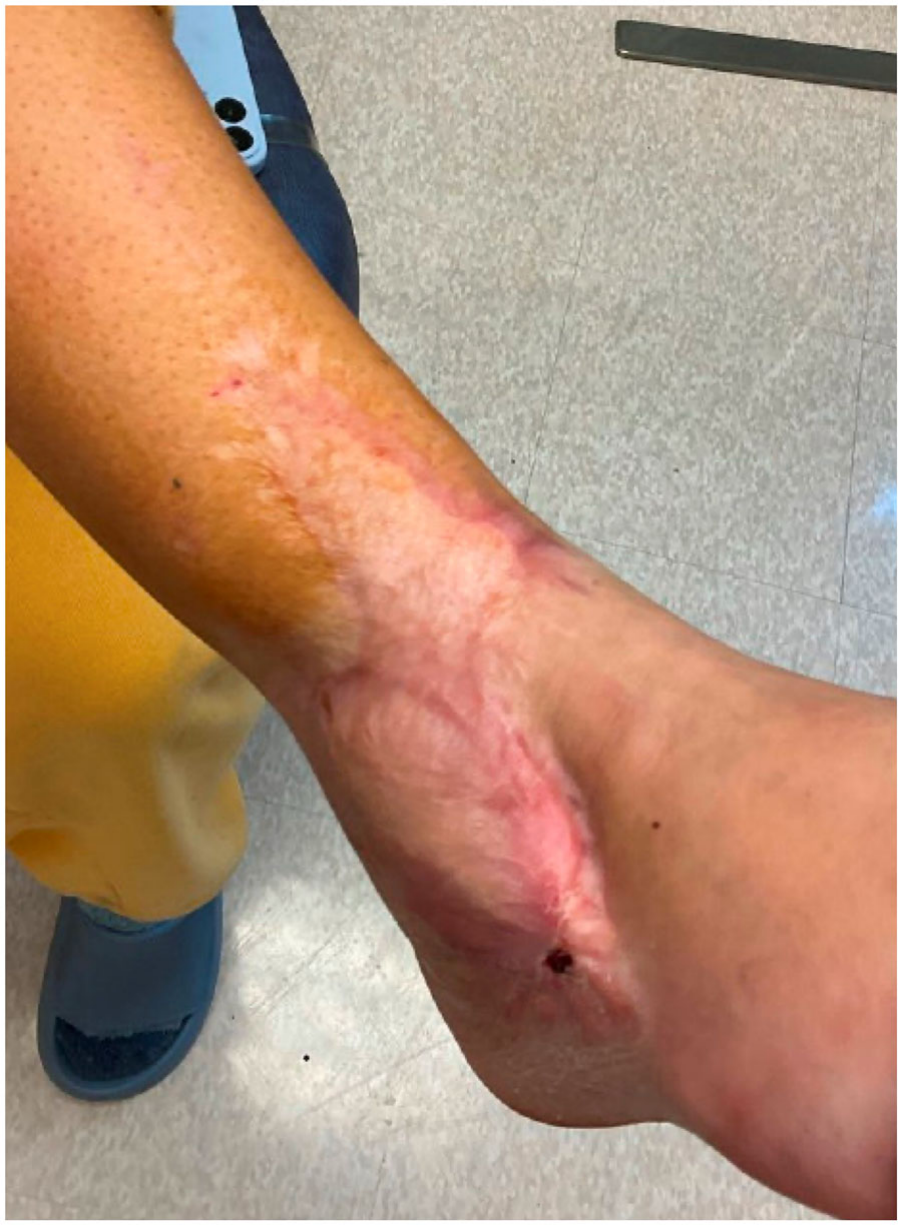
Right lower extremity 12-months postoperatively after flap salvage procedure.

Despite the artery and venae comitantes of the pedicle being resected to the level of healthy appearing intima, only weak flow through the artery was achieved. The patient’s systolic pressure was noted to be in the upper 80s mmHg, which was her baseline pressure. After increasing the systolic blood pressure to the high 90s mmHg, there was strong flow through the anterior tibial artery. The arterial and venous anastomoses were revised, and strong flow was demonstrated with a good Doppler signal, despite a bruised and congested appearance of the flap skin. The skin bridge over the pedicle was not closed to minimize external compression, and the resultant defect was covered with a dermal substitute and negative pressure wound therapy device (NPWT). Leech therapy was initiated on the exposed skin of the flap, and the patient was subsequently readmitted to the PICU, followed by initiation of the dangle protocol on postoperative day 14. The patient’s systolic blood pressure was maintained above 90 mmHg. Although there were areas of superficial epidermolysis, the flap remained perfused. The patient was discharged home three weeks postoperatively with regular follow-up for wound monitoring and NPWT changes. Split-thickness skin grafting was performed on postoperative day 28 with a complete skin graft take (Figure 3). At one-year follow-up, the flap remains healthy and viable, and the split-thickness skin graft is well healed (Figure 4).

## Discussion

Free flap tissue transfer is the standard for soft tissue reconstruction following many extremity oncological resection procedures. These are generally successful, with the overall rate of flap loss reported in some studies to be 5%, although the re-exploration rate may be up to 15% [3]. Generally, flap failure occurs within 72 h post-operatively and is largely due to vascular thrombosis, likely secondary to poor pedicle geometry or technical issues at the anastomotic site [2]. For those flaps requiring salvage within 72 h, the successful salvage rate was approximately 75% [3]. Yu and colleagues found the success rate of flap salvages changes dramatically after this post-operative time interval, dropping from 60% to 20% [4]. It is even rarer for vascular compromise to occur after 7 days post-operation, with one comprehensive review reporting less than 0.1% of all free flap failures occurring in the second week, the overwhelming majority of which completely fail [1].

The overall success rate of free tissue transfers to lower extremities is lower than other regions due to several factors such as gravity-driven edema in the leg, traumatic injury affecting the vessels, and abnormal or thrombogenic recipient vessels secondary to peripheral vascular disease [5]. It is critical to monitor patients that have risk factors for flap failure and consider postoperative protocols that may address lower extremity edema and congestion, such as progressive dangling.

In the normal limb, the response to an increase in capillary pressure from weight bearing and gravity is to increase pre-capillary vascular resistance *via* the venoarterial reflex [6]. However, due to the lack of appropriate sympathetic innervation in the post-surgical free flap, this compensatory mechanism is not viable, and the affected tissues are unable to adequately respond to an increase in capillary pressure [7]. This can lead to excess edema and ischemia, increasing the risk of flap failure. When executed correctly, a progressive dangling protocol can be a valuable technique to allow the flap to progressively adapt to the physiological constraints caused by congestion and better condition it to handle ischemia [7]. In our patient, venous congestion secondary to edema may have contributed to flap compromise. While a dangle protocol was initiated for our patient, it is possible that adherence to this protocol by less-experienced floor nursing staff was less strict than with PICU nursing staff who are more experienced in post-operative flap monitoring.

Another major factor driving increased rates of flap failure is associated with preoperative radiation therapy, which our patient had undergone. In a meta-analysis conducted by Herle et al. 2886 pre-operative irradiated flaps were compared with 3561 non-irradiated flaps from 24 studies. Analysis demonstrated statistically significant increased risks of flap failure (RR1.48, *p* = 0.003), flap complications (RR1.84, *p* < 0.001), reoperation (RR2.06, *p* < 0.001), and fistula formation (RR 2.05, *p* < 0.001) [8].

Due to the absence of any large clots in the main blood vessels during flap exploration, it is also possible that flap compromise was the result of microthrombi accumulation in circulation due to radiation-related injury – a phenomenon described by Singh and colleagues when evaluating microvascular complications post-radiation therapy [2]. The leading hypotheses explaining these complications are radiation-induced delayed neovascularization or an activated prothrombic state at the microvascular site.

Late flap failures between 7–14 days post-operation may be the result of delayed neovascularization, and thus new vessel formation in the recipient bed is insufficient to fully meet the requirements of the flap. Histologically, tissues undergoing radiation show a reduction in both the number and diameter of capillaries, thereby impacting vascularization in the transitional zone between the graft and irradiated tissue bed and causing vascular thrombosis and potential flap failure [9].

Radiation therapy has also been shown to induce a prothrombic and proinflammatory state by causing a higher incidence of vessel lesions. Formerly irradiated vessels display reduced smooth muscle density, increased vessel wall fibrosis and endothelial cell dehiscence, and enhanced coagulation and fibrinolysis [10]. In addition to structural changes, there is also increased expression of proinflammatory and prothrombotic cytokines, leukocyte adhesion molecules, and NF-kB in the post-radiation tissue bed [11].

While further studies are needed to better understand the influence that dosage and timing that radiation has on vessel damage, there is a clear and statistically significant risk for total or partial flap failure as a result of previous irradiation [11].While our patient did not appear to have gross intimal changes of her donor anterior tibial artery, it is possible that microscopic histological changes and proinflammatory chemical changes contributed to flow compromise.

Another potential contribution to flap compromise is an acute decrease in arterial flow secondary to arterial insufficiency. Intraoperatively, the patient was noted to have poor flow through the anterior tibial artery until her systolic pressure was increased. During and after the second surgery, the patient’s systolic pressure was maintained above 90 mmHg, which was not done in the week prior to flap failure.

In this report, we detail the case of a pediatric patient who experienced a late lower extremity free flap vascular compromise and risk of failure, along with successful flap salvage after revision anastomosis and infusion of Alteplase. Based on the patient’s clinical presentation and findings during flap exploration, it is possible that low systemic blood pressure or microthrombi accumulation, possibly as a result of radiation therapy, contributed to delayed flap compromise.
